# Improvement Effect and Mechanism of Hydroxytyrosol on Skin Aging Induced Advanced Glycation End Products

**DOI:** 10.3390/nu17172810

**Published:** 2025-08-29

**Authors:** Rui Fan, Yuxin Ma, Meng Sun, Haohao Zhang, Yaxin Han, Junbo Wang, Wenli Zhu, Zhaofeng Zhang

**Affiliations:** Department of Nutrition and Food Hygiene, School of Public Health, Peking University, Haidian District, Beijing 100191, China; fanruirf@bjmu.edu.cn (R.F.); 15733929487@163.com (Y.M.); m15910287861@163.com (M.S.); 2411210082@bjmu.edu.cn (H.Z.); hanyaxin@bjmu.edu.cn (Y.H.); wjbxybmu@bjmu.edu.cn (J.W.); zhuwenli@bjmu.edu.cn (W.Z.)

**Keywords:** skin aging, hydroxytyrosol, advanced glycation end products, inflammatory response, oxidative

## Abstract

**Objectives:** Skin aging, often accelerated by dietary advanced glycation end products (AGEs), poses both cosmetic and health challenges. This study explores the protective effects of hydroxytyrosol (HT), a potent antioxidant found in olives, against AGEs-induced skin aging in mice. **Methods:** A total of forty-eight 8-month-old specific pathogen-free (SPF) male C57BL/6J mice were randomly assigned to one of four groups: control, model, low-dose hydroxytyrosol (HT25), and high-dose hydroxytyrosol (HT50). An additional group of six 6-week-old SPF male C57BL/6J mice served as the youth group. The experimental period lasted 16 weeks. Following the intervention, skin, serum, and ileum samples were collected. **Results:** The results demonstrated that HT50 significantly increased skin moisture, epidermal thickness, and dermal thickness (*p* < 0.05). HT50 also significantly elevated hydroxyproline levels as well as superoxide dismutase (SOD) and glutathione peroxidase (GSH-Px) activities in the skin while reducing malondialdehyde (MDA) content (*p* < 0.05). Furthermore, HT50 significantly reduced the levels of interleukin-6 (IL-6), interleukin-1β (IL-1β), and tumor necrosis factor-α (TNF-α) (*p* < 0.05). Regarding intestinal integrity, hydroxytyrosol intervention (either HT25 or HT50) significantly increased the positive staining ratios of zonula occludens-1 (ZO-1) and occludin in the ileum (*p* < 0.05). **Conclusions:** HT improves skin hydration, thickness, and collagen levels while reducing oxidative stress and inflammation. Notably, HT also enhances intestinal barrier function, suggesting a role for the gut–skin axis. These findings highlight HT’s potential as a natural intervention for skin aging.

## 1. Introduction

As the body’s largest organ, the skin orchestrates vital physiological functions encompassing barrier integrity, thermoregulation, sensory transduction, metabolic regulation, and systemic homeostasis maintenance [1]. Skin aging constitutes both a biomarker and driver of organismal senescence, with emerging evidence revealing bidirectional linkages between cutaneous aging and multisystem degeneration [2]. Critically, aged skin serves as a visible indicator of systemic physiological decline. The pathophysiological cascade initiated by diminished epidermal regeneration, barrier compromise, and impaired wound healing in aging populations heightens susceptibility to dermatological infections while concurrently elevating risks for neurocognitive disorders and cardiovascular pathologies [3,4]. These interconnections underscore the imperative for mechanistic investigations of skin aging, which may yield critical insights for holistic health assessment, mortality prediction, and longevity research [5].

Clinically, skin aging manifests through wrinkle formation, pigmentary irregularities, xerosis, epidermal thinning, and diminished dermal elasticity alterations arising from synergistic intrinsic chronoaging and extrinsic environmental exposures [6]. Intrinsic aging represents an inevitable process governed by genetic and metabolic determinants, whereas extrinsic aging predominantly results from environmental insults, including airborne pollutants, tobacco by-products, dietary factors, and ultraviolet radiation [7]. Advanced glycation end products (AGEs), generated non-enzymatically via Maillard reactions between reducing sugars and proteins/lipids/nucleic acids, are classified as endogenous (metabolically derived) or exogenous (diet-sourced, particularly from thermally processed foods) [8,9,10].

Notably, accumulating evidence indicates that dietary AGEs substantially contribute to the systemic AGE pool, representing a significant source of total body AGE burden [11]. AGEs interact with and modify collagen and elastin fibers, diminishing skin elasticity and increasing rigidity. These effects are mechanistically linked to altered synthesis of extracellular matrix (ECM) components and dysregulation of enzymes like matrix metalloproteinases (MMPs), responsible for ECM turnover [12,13]. AGEs also promote fibroblast apoptosis, further compromising skin structural integrity. Concurrently, elevated AGE levels correlate with reduced dermal collagen content and increased fragmentation [14]. Furthermore, AGEs exacerbate oxidative stress by promoting reactive oxygen species (ROS) generation and inducing mitochondrial dysfunction [14,15]. This oxidative burden is amplified when key antioxidant enzymes, such as SOD, are inactivated by glycation [16]. AGEs also activate pro-inflammatory transcription factors, notably NF-κB, leading to increased release of cytokines such as TNF-α and IL-6, which accelerate skin aging [15]. An additional pathological mechanism involves AGE engagement with their receptor (RAGE), triggering signaling cascades that dysregulate processes, including proliferation, autophagy, apoptosis, and inflammatory responses [15,17]. Therefore, comprehensively understanding the mechanisms by which AGEs inevitably generated both endogenously and derived exogenously from the diet contributes to accelerated skin aging is essential for developing effective interventions. However, in-depth research specifically focused on AGE-induced skin aging and strategies for its amelioration remains notably scarce.

Evidence indicates that polyphenol-rich botanicals—administered topically or orally—can mitigate clinical manifestations of skin aging [18]. Hydroxytyrosol (HT), a secoiridoid polyphenol predominantly esterified in *Olea europaea* L. derivatives, demonstrates pleiotropic bioactivities, including antimicrobial, anti-inflammatory, antitumor, and potent antioxidant properties [19]. Its exceptional radical scavenging capacity derives from ortho-dihydroxyphenyl electron donation and phenoxyl radical stabilization through intramolecular hydrogen bonding [20]. HT attenuates UV-induced photodamage while enhancing cellular antioxidant defenses via Nrf2-mediated upregulation of phase II enzymes (including SOD and glutathione peroxidase, GPx), thereby suppressing ROS generation [21]. Furthermore, HT exerts anti-inflammatory effects by modulating cytokine production and inhibiting NF-κB signaling cascades [22,23]. Given this mechanistic profile, we postulated that HT may ameliorate AGE-associated skin aging.

To accurately model physiological conditions and dietary behaviors relevant to high AGE intake, this study employed middle-aged mice fed a high-AGE diet. Our objectives were to assess the efficacy of hydroxytyrosol in ameliorating skin aging induced by this diet and to elucidate its underlying mechanisms. The findings provide valuable insights for formulating safer nutritional strategies aimed at preventing and treating AGE-associated skin aging.

## 2. Materials and Methods

### 2.1. Chemicals

Hydroxytyrosol, with a purity of 98% as determined by high-performance liquid chromatography (HPLC), was procured from Shanghai McLean Biochemical Technology Co., Ltd. (Shanghai, China) (catalog number: H811041-5g, batch number: C10885750). The AIN-93G standard diet was obtained from Beijing Ke’ao Xieli Feed Co., Ltd. (Beijing, China) (license number: SCXK2019-0003).

### 2.2. Experimental Animals and Treatment

**Figure 1:**
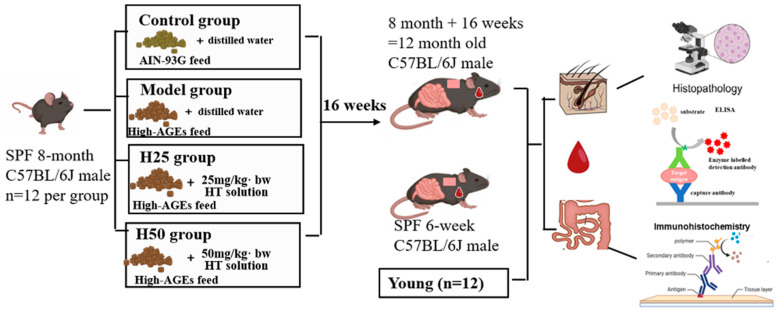
The flow of mouse treatments.

A total of 48 8-month-old male C57BL/6J mice (specific pathogen-free, SPF) were designated as the experimental cohort, with six-week-old SPF male C57BL/6J mice serving as young controls (n = 12/group). All animals were sourced from Charles River Laboratories and maintained in individual ventilated cages under standardized conditions (22 ± 1 °C, 55 ± 10% humidity, 12:12 light-dark cycle), with ad libitum access to food and water. Following a 72 h acclimatization period, randomization was achieved by generating random numbers using a computer and then grouping them into four groups accordingly. And interventions commenced according to the regimen detailed in Figure 1. The hydroxytyrosol (HT) dosage (25 or 50 mg/kg/day) was established from previous pharmacokinetic studies [24]. After 16 weeks of intervention, the blood was collected by retro-orbital (orbital sinus) blood collection, and then, cervical dislocation was carried out. The blood collection and euthanasia were detailed as follows: (i) mice were placed in a clean, transparent induction chamber connected to a precision vaporizer; (ii) the mice were gently induced with 3–4% isoflurane in oxygen until they lost consciousness, as verified by the absence of the pedal reflex (toe pinch); (iii) once the mice were unconscious and unresponsive, each was promptly removed from the chamber; (iv) the anesthetized mouse was then transferred from the chamber, and the blood collection procedure was performed promptly; (v) cervical dislocation was performed as a secondary physical method to ensure death. This technique was executed by trained personnel who applied firm, rapid traction at the base of the skull, resulting in immediate dislocation of the vertebrae and severance of the spinal cord; and (vi) death was confirmed by the absence of a heartbeat and respiration, along with fixed and dilated pupils. After euthanasia, the dorsal skin and ileal specimens were surgically excised. After euthanasia, the dorsal skin and the ileal specimens were collected, and tissue aliquots were either immediately processed for histopathological analysis or flash-frozen in liquid nitrogen for cryopreservation at −80 °C pending immunoblotting. All experimental protocols received prior approval by the Peking University Biomedical Ethics Committee (LA2023500, approved on 10 October 2023).

### 2.3. High-AGEs Feed Preparation

The high-AGE diet was sourced from Beijing Botai Hongda Biotechnology Co., Ltd. (Beijing, China) (SCXK2020-0004). Preparation involved supplementing base AIN-93G powder with a double-concentration vitamin mixture. This supplemented formulation was pelleted and heat-processed at 128 °C for 3 h to induce glycation. Table 1 details the nutritional profile and AGEs (*N-*ε-carboxymethyl lysine, CML) content before and after thermal processing.

### 2.4. General Situation of Animals

First, we checked and recorded any adverse events every day. Mouse coat condition, body weight, food consumption, water consumption, and fecal output were monitored weekly. Additionally, the weights of both remaining feed and newly provided feed were recorded each week. Food intake was calculated as the difference between the feed provided and the residual feed. Once it is detected that the weight of any mouse drops by more than 20% or its food intake decreases by 50%, the experiment should be immediately stopped.

### 2.5. Determination of Skin Moisture Content

A skin specimen, measuring approximately 2 × 2 cm^2^, was excised from the dorsal region of the mice. Subsequent to excision, subcutaneous fat, connective tissues, and the epidermis were meticulously removed. The wet mass of the skin was accurately determined using an analytical balance. Five specimens were then subjected to a drying process in an oven maintained at 80 °C for a duration of 24 h, after which the dry mass was recorded. The moisture content of the skin was calculated using the following formula:

Moisture Content (%) = [(Wet Mass − Dry Mass)/Wet Mass] × 100.

### 2.6. Histopathological Evaluation of Skin Tissue

Post-euthanasia, dorsal skin specimens were excised, and selected five mice from each group were immersion-fixed in 4% paraformaldehyde (PFA) for 24 h at 4 °C. Fixed tissues underwent standardized histoprocessing: trimming, graded ethanol dehydration, xylene clearing, paraffin embedding, and sectioning at 5 μm thickness. Sections were stained with hematoxylin and eosin (H&E) and then cover-slipped using permanent mounting medium. Digital histomorphometry was performed using a Nikon Eclipse Ci-L microscope (Nikon Precision (Shanghai) Co., Ltd., Shanghai, China) equipped with a DS-Fi3 camera (Nikon Precision (Shanghai) Co., Ltd., Shanghai, China) under Köhler-illuminated conditions at 100× magnification. Epidermal and dermal thickness measurements were obtained at five randomized, non-overlapping fields per section using Image-Pro Plus 6.0 software calibrated with a stage micrometer. Mean thickness values were derived for statistical analysis.

### 2.7. Measurement of Skin Hydroxyproline (HYP) Levels

Hydroxyproline (HYP) content—a collagen-specific biomarker—was quantified in dorsal skin tissue using assay kits (Nanjing Jiancheng Bioengineering Institute, Nanjing, China) following alkaline hydrolysis. The enzyme-linked immunosorbent assay (ELISA) protocol comprised:

(1) Coating: 96-well plates were coated with 100 μL/well antigen/antibody solution in carbonate-bicarbonate buffer (pH 9.6), incubated overnight at 4 °C.
(2) Washing: Wells were washed 3–5 times with phosphate-buffered saline containing 0.05% Tween-20 (PBST), with residual liquid removed by aspiration followed by plate inversion on absorbent paper.
(3) Blocking: 200 μL blocking buffer (5% bovine serum albumin in PBST) was added per well, incubated for 1 h at room temperature (RT, 22 ± 2 °C).
(4) Standards/Samples: Serial dilution standards and tissue homogenates (prepared in RIPA buffer) were added to wells and incubated for 2 h at RT.
(5) Detection Antibody: Wells received 100 μL horseradish peroxidase-conjugated detection antibody (1:2000 in blocking buffer), incubated for 1 h at RT.
(6) Substrate Reaction: 100 μL tetramethylbenzidine (TMB) substrate was added per well and incubated for exactly 30 min at RT in darkness. Reactions were terminated with 50 μL 2 M H_2_SO_4_.
(7) Absorbance Measurement: Optical density at 450 nm (reference 630 nm) was measured within 30 min using a microplate reader (BioTek Synergy H1). HYP concentrations of seven mice in each group were calculated using four-parameter logistic regression against the standard curve.

### 2.8. Evaluation of Oxidative Stress Markers

Seven mice in each group were carried out with ELISA assay. Catalase (CAT) activity was quantified using the ammonium molybdate spectrophotometric assay, which measures residual hydrogen peroxide through yellow complex formation at 405 nm. Superoxide dismutase (SOD) activity was determined by the hydroxylamine method, based on the inhibition of nitrite formation from hydroxylamine oxidation at 550 nm. Glutathione peroxidase (GSH-Px) activity was assessed spectrophotometrically by monitoring NADPH oxidation at 340 nm. Malondialdehyde (MDA) levels were measured via the thiobarbituric acid reactive substances (TBARS) assay at 532 nm. All assay kits were procured from Nanjing Jiancheng Bioengineering Institute (China), with strict adherence to manufacturer’s protocols throughout analyses.

### 2.9. Measurement of Serum Inflammatory Cytokines

Seven mice in each group were carried out through the measure of inflammatory indicators. Serum concentrations of interleukin-6 (IL-6), interleukin-1β (IL-1β), and tumor necrosis factor-α (TNF-α) were quantified using enzyme-linked immunosorbent assay (ELISA) according to manufacturer specifications. Commercial ELISA kits (Invitrogen, Waltham, MA, USA) were employed with minimum detectable limits of 8 pg/mL for IL-1β, 4 pg/mL for IL-6, and 8 pg/mL for TNF-α. All assays were performed in strict accordance with the provided protocols, including standard curve generation and quality control measures.

### 2.10. Intestinal Morphology and Barrier Function Evaluation

Ileal segments were harvested postmortem, and a selected five mice from each group were immersion-fixed in 10% neutral buffered formalin (NBF) for 24 h at 4 °C. Tissues underwent cryopreservation via flash-freezing in liquid nitrogen for histopathological and immunohistochemical (IHC) analyses. Tight junction proteins zonula occludens-1 (ZO-1) and occludin were immunolocalized using primary antibodies: rabbit anti-ZO1 polyclonal antibody (Abcam ab96587; Cambridge, UK) and anti-occludin monoclonal antibody (clone E6B4R; Cell Signaling Technology #13409S; Danvers, MA, USA). Antigen retrieval was performed in citrate buffer (pH 6.0), followed by blocking with 5% normal goat serum. Immunolabeled sections were quantitatively analyzed using ImageJ v1.53a with threshold-based segmentation. The immunopositive area fraction (%) was calculated to evaluate intestinal barrier integrity. Groupwise comparisons were analyzed by one-way ANOVA with Tukey’s post hoc test.

### 2.11. Statistical Analysis

Statistical analyses were conducted utilizing SPSS software version 22 (SPSS Inc., Chicago, IL, USA). The experimental results are presented as mean ± standard deviation or median (interquartile range). Homogeneity of variance was assessed using one-way ANOVA. Between-group comparisons utilized Fisher’s least significant difference (LSD). For data exhibiting non-normality or heteroscedasticity, appropriate variable transformations were applied to satisfy the assumptions of normality and homogeneity of variance. If these transformations did not achieve the required assumptions, non-parametric tests were employed for statistical analysis. Pairwise comparisons between the experimental and control groups were conducted using Holm–Bonferroni method, with a significance threshold set at *p* < 0.05.

## 3. Results

### 3.1. General Situation of Animals

#### Changes in the Body Weight and Food Intake of Mice

There are no adverse events during the intervention period. Body weight serves as a critical health indicator in experimental animals. During the 16-week intervention, we systematically monitored physiological parameters across all cohorts, with particular emphasis on longitudinal body weight changes and dietary intake patterns.

**Figure 2:**
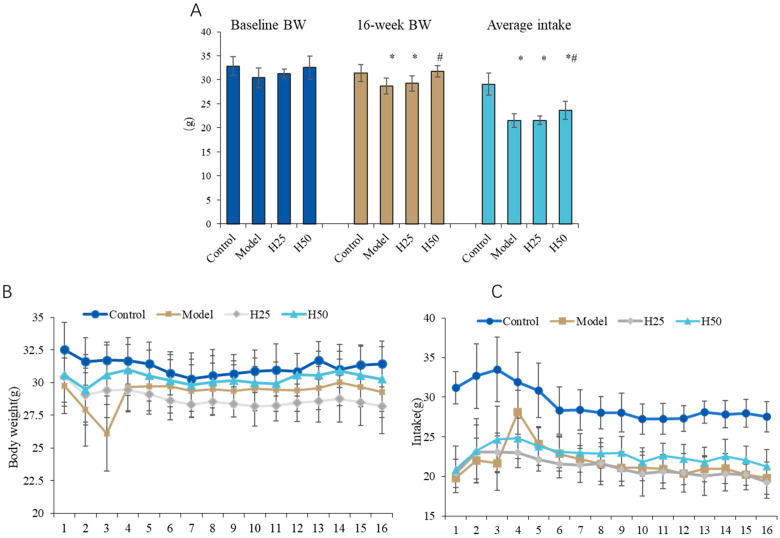
The body weights and feed intake in the different groups. (**A**) The change of weights and intake; (**B**) body weight; (**C**) food intake. Statistical analysis performed using one-way ANOVA with LSD post hoc test; * compared with the model group, *p* < 0.05, # compared with the control group, *p* < 0.05.

As shown in Figure 2A, no significant between-group differences in baseline body weights were observed (*p* > 0.05). After 16 weeks, control animals exhibited significantly higher terminal body mass (31.44 ± 1.76 g) compared to the AGE model group (28.71 ± 1.69 g; *p* < 0.05), suggesting that chronic AGE exposure may contribute to weight reduction. Notably, high-dose hydroxytyrosol (H50) intervention significantly attenuated this weight loss (29.83 ± 1.54 g vs. model; *p* < 0.05), indicating protective efficacy against AGE-induced metabolic alterations (Figure 2B).

Consistent with weight observations, dietary analysis (Figure 2C) revealed significantly reduced cumulative food intake in the model group (21.56 ± 1.41 g/week) versus controls (29.11 ± 2.29 g/week; *p* < 0.05). The H50 group showed a significant increase in intake (23.66 ± 1.87 g/week; *p* < 0.05 vs. model).

### 3.2. The Improvement Effect of Hydroxytyrosol on Skin Aging in Mice

#### 3.2.1. Histopathological Evaluation of Skin Tissue

**Figure 3:**
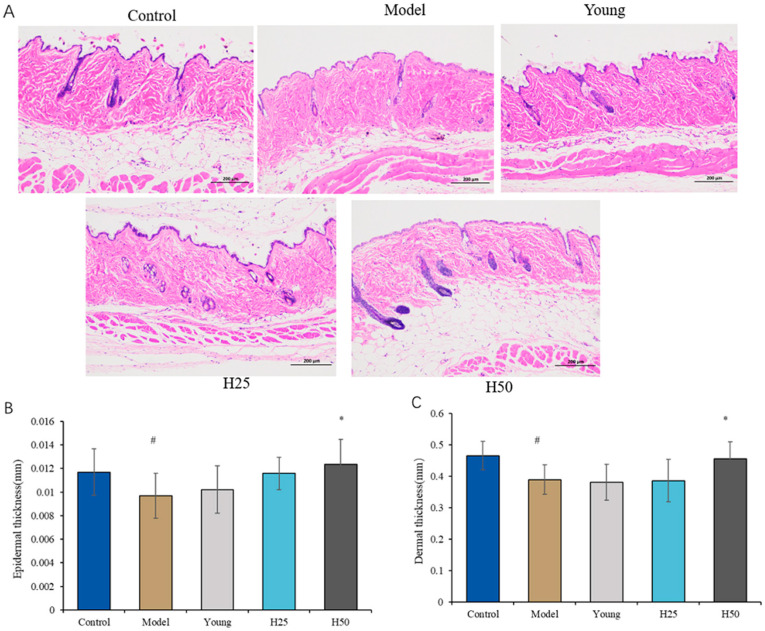
The skin structural in different groups. (**A**) H&E staining of the skin (light microscope, scale bar 200 μm); (**B**) epidermal thickness; (**C**) dermal thickness. Statistical analysis performed using Kruskal–Wallis test with Holm–Bonferroni for pairwise comparison; results are expressed as median (interquartile range). Statistical significance is indicated as * *p* < 0.05 (compared with the model group), # *p* < 0.05 (compared with the control group).

Structural alterations in skin tissue, particularly epidermal and dermal thickness, were evaluated through hematoxylin and eosin (H&E) staining. As shown in Figure 3A, mice fed the advanced glycation end product-enriched diet (AGE model group) exhibited significant epidermal and dermal thinning relative to controls, demonstrating AGE-induced skin atrophy. Conversely, high-dose HT intervention substantially restored epidermal and dermal architecture.

Quantitative morphometric analysis (Figure 3B,C) confirmed significantly reduced median epidermal thickness [0.009 (0.002) mm vs. 0.012 (0.002) mm, *p* < 0.05) and dermal thickness (0.389 (0.045) mm vs. 0.465 (0.045) mm; *p* < 0.05] in the model group versus controls. While HT intervention showed dose-dependent protective effects, only high-dose HT significantly increased both epidermal thickness [0.0123 (0.002) mm; *p* < 0.05] and dermal thickness [0.455 (0.054) mm; *p* < 0.05] relative to the model group. These findings demonstrate that high-dose HT effectively counteracts AGE-induced skin atrophy through structural preservation of cutaneous layers.

#### 3.2.2. Determination of Skin Moisture Content and Hydroxyproline (HYP) Levels

**Figure 4:**
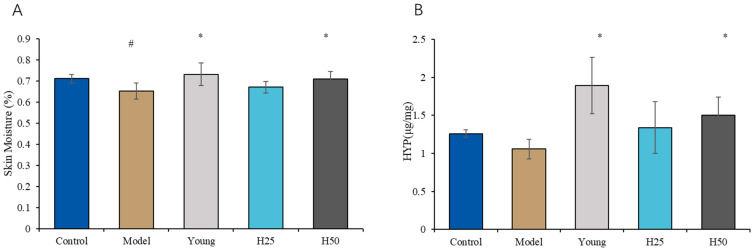
The changes in skin moisture and hydroxyproline in mice of different groups. (**A**) Skin moisture; (**B**) hydroxyproline. Statistical analysis performed using one-way ANOVA with LSD post hoc test; results are expressed as mean ± SD. Statistical significance is indicated as * *p* < 0.05 (compared with the model group), # *p* < 0.05 (compared with the control group).

As shown in Figure 4A, the AGE model group exhibited significantly reduced cutaneous hydration status (65.20 ± 3.73%) versus controls (71.00 ± 1.95%; *p* < 0.05), indicating AGE-mediated impairment of epidermal barrier function. High-dose hydroxytyrosol (H50) intervention significantly restored hydration parameters relative to the model group (70.94 ± 3, 53%; *p* < 0.05). Although low-dose HT (H25) showed a non-significant elevation in hydration (66.97 ± 2.65%; *p* = 0.072), only H50 demonstrated complete restoration. These findings establish a dose-dependent therapeutic effect of HT on glycation-induced cutaneous dehydration.

Hydroxyproline (HYP), a collagen-specific amino acid critical for triple-helix stabilization via post-translational hydroxylation, serves as a biochemical marker of collagen integrity. Reduced dermal HYP content reflects accelerated collagen degradation and is recognized as a molecular hallmark of skin aging. As quantified in Figure 4B, while HYP levels in the AGE model group (1.057 ± 0.128 μg/mg) showed no significant difference versus controls (1.260 ± 0.051 μg/mg; *p* = 0.062), this trend toward reduction—when contextualized with significant epidermal thinning (Figure 3) and compromised hydration (Figure 4A)—collectively confirms successful induction of glycation-accelerated skin aging. Importantly, high-dose hydroxytyrosol (H50) intervention significantly elevated HYP content (1.506 ± 0.233 μg/mg; *p* < 0.05 vs. model), demonstrating collagen-preserving efficacy against AGE-mediated damage.

#### 3.2.3. Evaluation of Oxidative Stress Markers

**Figure 5:**
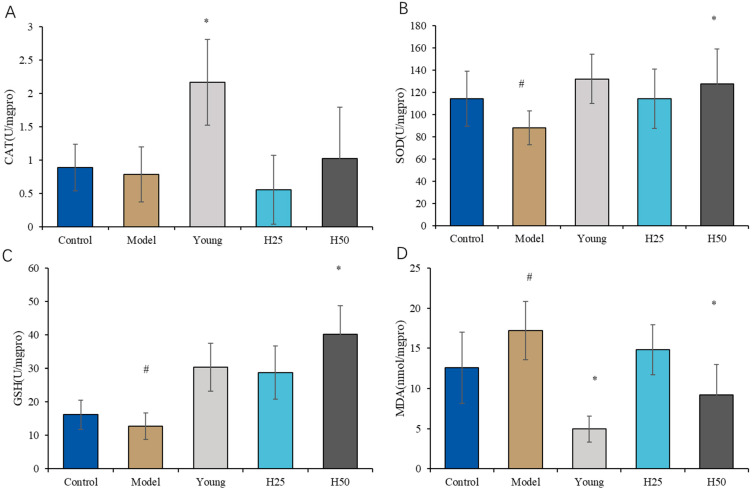
The oxidative stress in the different groups. (**A**) CAT; (**B**) SOD; (**C**) GSH-Px; (**D**) MDA. Statistical analysis performed using one-way ANOVA with LSD post hoc test; results are expressed as mean ± SD. Statistical significance is indicated as * *p* < 0.05 (compared with the model group), # *p* < 0.05 (compared with the control group).

As shown in Figure 5A, catalase (CAT) activity in the AGE model group was reduced relative to both young and high-dose hydroxytyrosol (H50) groups, though only the model-versus-young comparison reached statistical significance (*p* < 0.05).

Figure 5B demonstrates significantly suppressed superoxide dismutase (SOD) activity in the model group versus controls (*p* < 0.05), which was effectively restored by H50 intervention (*p* < 0.05 vs. model). Similarly, glutathione peroxidase (GSH-Px) activity (Figure 5C) showed significant reduction in the model group (*p* < 0.05 vs. control), with H50 normalizing activity to control levels (*p* < 0.05 vs. model). These findings indicate that dietary AGEs impair key antioxidant defenses (SOD/GSH-Px), while high-dose HT counteracts glycative stress through enzymatic potentiation.

Lipid peroxidation analysis (Figure 5D) revealed significantly elevated malondialdehyde (MDA) levels in the model group versus controls (*p* < 0.05). High-dose HT (H50) intervention significantly attenuated MDA accumulation (*p* < 0.05 vs. model), indicating reduced oxidative membrane damage. Mechanistically, HT likely mediates these effects through enhanced ROS scavenging and membrane stabilization. While low-dose HT (H25) showed non-significant trends toward improved SOD (*p* = 0.12) and GSH-Px (*p* = 0.08) activities, only H50 achieved statistical significance, confirming dose-dependent efficacy against AGE-induced oxidative pathology.

### 3.3. Measurement of Serum Inflammatory Cytokines

**Figure 6:**
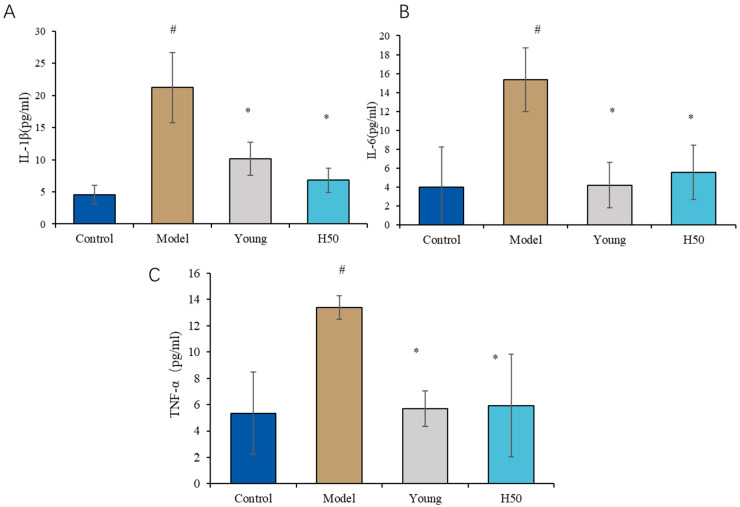
The serum inflammatory cytokines in the different groups. (**A**) IL-1β; (**B**) IL-6; (**C**) TNF-α. Statistical analysis performed using one-way ANOVA with LSD post hoc test. Results are expressed as mean ± SD. Statistical significance is indicated as * *p* < 0.05 (compared with the model group), # *p* < 0.05 (compared with the control group).

As shown in Figure 6A–C, mice fed AGE-enriched diet exhibited significantly elevated serum concentrations of pro-inflammatory cytokines IL-6 (4.7-fold increase), IL-1β (3.8-fold), and TNF-α (2.5-fold) compared to controls (*p* < 0.05). Both low-dose (H25) and high-dose (H50) hydroxytyrosol interventions significantly attenuated these elevations, reducing cytokine levels to near-baseline values (*p* < 0.05 vs. AGE model group). These findings demonstrate that hydroxytyrosol effectively suppresses AGE-induced systemic inflammation through dose-dependent inhibition of key pro-inflammatory mediators.

### 3.4. Cytokines Intestinal Morphology and Barrier Function Evaluation

**Figure 7:**
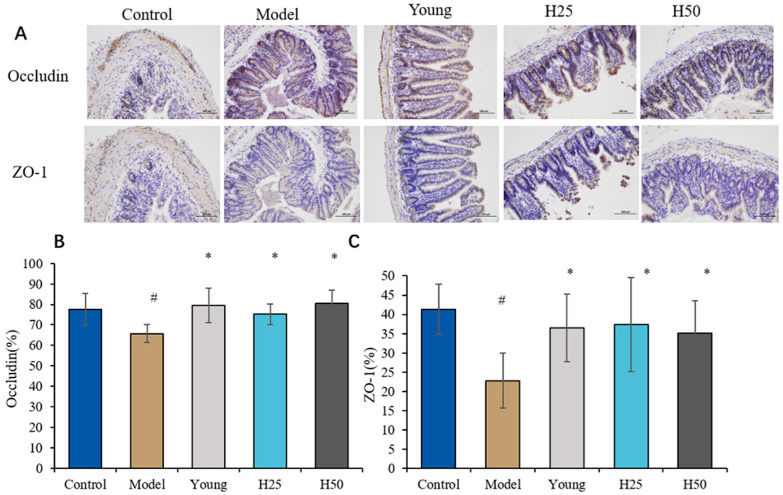
The intestinal morphology and barrier in the different groups. (**A**) Intestinal immunohistochemical staining of occludin protein and ZO-1 protein (light microscope, scale bar 100 μm); (**B**) ZO-1 protein expression; (**C**) occludin protein expression. Statistical analysis performed using one-way ANOVA with LSD post hoc test. Results are expressed as mean ± SD. Statistical significance is indicated as * *p <* 0.05 (compared with the model group), ^#^ *p* < 0.05 (compared with the control group).

Figure 7A presents representative immunohistochemical staining of intestinal tight junction proteins occludin and zonula occludens-1 (ZO-1). Semi-quantitative analysis categorized staining intensity as weak positivity (light yellow, low expression), moderate positivity (brown-yellow), and strong positivity (brown, high expression). Compared to controls, the AGE model group exhibited reduced moderate-to-strong staining, indicating significant downregulation of both proteins.

Quantitative analysis (Figure 7B) confirmed significantly decreased occludin-positive cells in the model group versus controls (65.8% ± 4.5% vs. 77.5% ± 7.8%; *p* < 0.05), demonstrating AGE-induced tight junction impairment. Both low-dose (H25: 74.3% ± 5.0%) and high-dose hydroxytyrosol (H50: 80.6% ± 6.3%) significantly restored occludin expression versus model (*p* < 0.05). Similarly, ZO-1-positive cells (Figure 7C) were significantly reduced in the model group (22.8% ± 7.1% vs. control 41.3% ± 6.6%; *p* < 0.05), with both HT interventions normalizing expression to control-equivalent levels (H25: 37.3% ± 12.2%; H50: 35, 2% ± 8.4%; *p* < 0.05 vs. model).

## 4. Discussion

This study addresses skin aging pathophysiology and potential therapeutic interventions. We established a novel in vivo model of diet-accelerated cutaneous aging by administering a high advanced glycation end product (AGE) diet to middle-aged mice, accurately recapitulating human middle-aged physiology and dietary patterns. While building upon established models [25,26], our approach represents the first dedicated application to cutaneous aging research. Given that skin aging arises from synergistic intrinsic processes and extrinsic factors—with dietary glycation being a principal accelerator in modern nutrition—this model enables development of targeted anti-aging strategies. Through comprehensive characterization of the aging phenotype, we evaluated HT’s role on preserving skin architecture and collagen homeostasis, which might be due to oxidative stress attenuation, anti-inflammatory effects, and gut–blood barrier modulation.

Skin hydration status and barrier integrity serve as critical biomarkers of dermatological health. Our results demonstrate significantly compromised skin homeostasis in the AGE model group, evidenced by reduced hydration (65.20% ± 3.73% vs. control 71.00% ± 1.95%; *p* < 0.05), epidermal thinning [0.009 (0.002) mm vs. control 0.012 (0.002) mm; *p* < 0.05], and dermal atrophy [0.389 (0.045) mm vs. control 0.465 (0.045) mm; *p* < 0.05] (Figure 2 and Figure 3). These findings align with established mechanisms of AGE-induced impairment of aquaporin-mediated water retention [27], thereby validating the physiological relevance of our dietary model for skin aging research. Notably, high-dose hydroxytyrosol (H50) intervention restored hydration parameters to near-normal levels (70.94% ± 3.53% vs. control 71.03% ± 1.95%; *p* > 0.05), demonstrating significant enhancement of stratum corneum water-binding capacity and barrier resilience. This moisturizing efficacy parallels the cutaneous effects observed with epigallocatechin gallate (EGCG) [28], suggesting shared polyphenolic mechanisms of barrier protection.

Skin aging is pathologically characterized by attenuated fibroblast function, diminished collagen/elastin biosynthesis, and progressive epidermal/dermal atrophy [29]. Histopathological analysis confirmed significant reductions in epidermal [0.009 (0.002) mm vs. 0.012 (0.002) mm; *p* < 0.05] and dermal thickness [0.389 (0.045) mm vs. 0.465 (0.045) mm; *p* < 0.05] in the AGE model group versus controls. High-dose hydroxytyrosol (HT) intervention reversed these structural deficits, restoring integumentary thickness to near-baseline levels (epidermal: 0.012 (0.002) mm; dermal: 0.455 (0.053) mm; *p* < 0.05)—an effect paralleled in studies of caffeic acid and cacao polyphenols [30,31]. Mechanistically, chronological aging reduces basal keratinocyte proliferation, leading to (1) epidermal thinning, (2) diminished dermal–epidermal junction surface area, and (3) compromised nutrient exchange that further impairs regenerative capacity [32]. Contrastingly, photoaging induces paradoxical epidermal hyperplasia through distinct pathways [33]. Concurrently, aged fibroblasts exhibit reduced collagen I/III synthesis, compromising extracellular matrix (ECM) integrity through weakened intercellular adhesion and cytoskeletal disorganization [34]. Crucially, AGE–RAGE binding activates mitogen-activated protein kinase (MAPK) pathways, triggering nuclear translocation of activator protein 1 (AP-1). This transcription factor upregulates matrix metalloproteinases (MMPs) that proteolytically degrade collagen IV and VII within the basement membrane, ultimately destabilizing dermal microstructure [35].

Our observations demonstrate that the hydroxyproline (HYP) content in the H50 group was significantly elevated compared to the model group. This finding aligns with the established role of HT in enhancing collagen synthesis and preserving extracellular matrix (ECM) integrity, as depicted in Figure 4B. Interestingly, no statistically significant difference in HYP content was observed between the model group and the control group, which is consistent with previous studies [36,37]. This may be attributed to the conditions of advanced glycation end products (AGEs) induction, such as a shorter duration or younger age of subjects compared to other reported conditions [25,38], which primarily affect collagen quality, including crosslinking and stability [39,40]. Indeed, discrepancies in hydroxyproline content between aging models and normal groups have been noted in prior research. According to Pu (2023) [36], the skin aging mouse model was established by exposing mice to D-galactose combined with UV radiation for 50 days. The hydroxyproline content in the skin of both the model group and the normal control group exhibited a decrease. However, as the induction period progressed, the rate of decrease in the model group decelerated. By the conclusion of the 50-day induction period, the hydroxyproline content in the skin of the model group remained lower than that of the normal control group, yet this difference was not statistically significant [36]. This observation is consistent with findings reported by Liu et al. (2014) [37]. Conversely, other studies have reported a significantly lower hydroxyproline content in the model group compared to normal mice [41], while some studies have even found it to be higher [42].

Advanced glycation end products (AGEs) potentiate oxidative stress and inflammation through three primary mechanisms: (i) covalent crosslinking of extracellular matrix proteins and intracellular targets; (ii) generation and accumulation of reactive oxygen species (ROS); and (iii) receptor-mediated activation, particularly via the receptor for AGEs (RAGE), triggering pathogenic signaling cascades [43]. The predominant skin damage pathway initiates with AGE–RAGE binding, subsequently activating PI3K/Akt, MAPK/ERK, and JAK2/STAT1 signaling axes. This leads to nuclear translocation of transcription factors NF-κB and FOXO1, upregulating pro-inflammatory (TNF-α, IL-6, and IL-1β) and pro-apoptotic mediators (caspase-3, Bax) [44]. Chronic low-grade inflammation represents a hallmark of AGE-accelerated aging. Our data confirm significantly elevated serum pro-inflammatory cytokines in the model group versus controls (IL-6: 3.8-fold; TNF-α: 2.5-fold; IL-1β: 4.7-fold; *p* < 0.05). Hydroxytyrosol intervention (both HT25 and HT50) effectively suppressed these cytokines (*p* < 0.05 vs. model), consistent with prior reports of polyphenolic anti-inflammatory efficacy [45,46].

Specific receptors for advanced glycation end products (AGEs), particularly the receptor for AGEs (RAGE), are expressed on multiple cell types. AGE–RAGE binding activates NADPH oxidase (NOX4 isoform), triggering reactive oxygen species (ROS) generation [47]. This oxidative cascade represents a central mechanism in AGE-induced skin aging. Our data demonstrate significant reductions in antioxidant enzyme activities in the model group versus controls: SOD, 88.19 ± 15.07 vs. 114.22 ± 24.73 U/mg protein, *p* < 0.05; CAT, 0.787 ± 0.41 vs. 0.890 ± 0.35 U/mg, *p* < 0.05, and GSH-Px: 12.73 ± 3.95 vs. 16.20 ± 4.3 U/mg, *p* < 0.05. Concurrently, MDA levels increased significantly 17.21 ± 3.64 vs. 12.59 ± 4.41 nmol/mg; *p* < 0.05), confirming AGE-mediated oxidative imbalance. High-dose hydroxytyrosol (H50) intervention restored antioxidant defenses (SOD: 127.53 ± 31.53; CAT: 1.02 ± 0.77; GSH-Px: 40.21 ± 8.53 U/mg) and reduced MDA (9.195 ± 3.8 nmol/mg) to near-normal levels (*p* < 0.05 vs. model). This demonstrates HT’s dual capacity for direct free radical scavenging and Nrf2-mediated enhancement of endogenous antioxidant systems [48]. Collectively, these findings establish that HT ameliorates AGE-induced skin aging primarily through attenuation of inflammatory pathways and oxidative stress, aligning with established polyphenolic mechanisms [45,49].

Furthermore, we investigated the role of the gut–skin axis and demonstrated that AGEs adversely affect gut epithelial barrier integrity, as evidenced by reduced expression of tight junction proteins ZO-1 and occludin (Figure 7). Upon epithelial injury, danger signal molecules are released [50], which is associated with an observed increase in inflammatory cytokine levels (Figure 6). Quantitative analysis revealed significantly reduced occludin (65.77% ± 4.51% vs. control 77.46% ± 7.90%; *p* < 0.05) and ZO-1 expression (22.78% ± 7.12% vs. 41.27% ± 6.59%; *p* < 0.05) in the model group, confirming AGE-induced intestinal compromise. The detrimental effects of AGEs on the human gut were further corroborated by experiments using small intestine organ cultures. These experiments revealed early alterations in the human intestinal mucosa upon AGEs stimulation, characterized by an increased number of CD25+ mononuclear cells and an elevated proliferation rate of crypt cells, which are biomarkers of intestinal mucosal inflammation and damage, respectively [51]. Hydroxytyrosol intervention dose-dependently restored tight junction protein expression (ZO-1: H50 35.17% ± 8.40%; occludin: H50 80.62% ± 6.27%; *p* < 0.05 vs. model), demonstrating HT’s capacity to preserve intestinal barrier function, reduce endotoxemia, and indirectly attenuate skin aging through gut-skin axis modulation.

This 4-month interventional study examined hydroxytyrosol supplementation in C57BL/6J mice for mitigating skin aging induced by dietary AGEs. Our investigation focused on cutaneous phenotypes and collagen metabolism while concurrently evaluating serum inflammatory markers and intestinal barrier integrity. We specifically elucidated HT’s mechanism of action against glycative skin damage through the gut–skin axis, emphasizing intestinal physical barrier function. Our findings align with reports that epigallocatechin gallate (EGCG) reduces systemic inflammation via enhanced intestinal barrier integrity, thereby secondarily supporting skin health [28]. Nevertheless, this study has several limitations: (i) mechanistic focus restricted to physical rather than biological/immune barriers; (ii) undetermined persistence of effects post-intervention; and (iii) exclusive reliance on murine models. Therefore, the future research, including the specific mechanisms of the skin–gut axis, particularly focusing on the biological and immune barriers, such as microbiome, should be explored. Additionally, a finding that warrants further validation through population studies will be verified. And importantly, further evaluating whether HT’s benefits persist after cessation should be considered.

## 5. Conclusions

This study demonstrates that hydroxytyrosol (HT) significantly ameliorates skin aging induced by dietary advanced glycation end products (AGEs), as evidenced by the restoration of skin hydration (an increase of 5.74% compared to the model group, *p* < 0.05), elevated hydroxyproline content (an increase of 42.5% compared to the model, *p* < 0.05), and increased dermal (0.066 mm) and epidermal (0.0027 mm) thickness (cv. model, *p* < 0.05). Mechanistically, HT exerts these effects through modulation of the gut–skin axis, enhancing intestinal barrier integrity (with ZO-1 expression increasing by 12.38% and occludin expression by 14.85% compared to the model, *p* < 0.05) while attenuating systemic inflammation (with reductions in IL-6, IL-1β, and TNF-α levels by 4.69-fold, 3.83-fold, and 2.50-fold, respectively, compared to the model, *p* < 0.05) and reducing oxidative stress (with SOD levels increasing by 44.6%, GSH-Px by 200%, and MDA levels decreasing by 46.6% compared to the model, *p* < 0.05). These findings establish HT’s dual role in barrier restoration and redox homeostasis, providing mechanistic foundations for evidence-based nutraceutical strategies targeting glycative skin aging.

## Figures and Tables

**Table 1:** Content of macronutrients, energy, and CML in feed before and after heating (per 100 g).

Ingredients	Before Baking	After Baking	*F*	*p*
Energy (kJ)	1576 ± 145	1760 ± 189	1.790	0.252
Crude protein (%)	17.82 ± 2.23	18.68 ± 1.87	0.262	0.636
Crude fat (%)	7.00 ± 1.11	7.73 ± 1.23	0.582	0.488
Carbohydrates (%)	64.31 ± 5.98	68.12 ± 6.75	0.535	0.505
CML (mg)	0.02 ± 0.01	0.14 ± 0.05 *	16.615	0.015

Statistical analysis performed using one-way ANOVA; * compared with the before baking, *p* < 0.05.

## Data Availability

The data are not publicly available due to privacy. The original contributions can be directed to the corresponding author.

## Abbreviations

The following abbreviations are used in this manuscript:

AGES	Advanced Glycation End Products
β-actin	Beta-action
CAT	Catalase
CML	Carboxy Methyl Lysine
ECM	Extracellular Matrix
GSH-Px	Glutathione Peroxidase
HYP	Hydroxyproline
HT	Hydroxytyrosol
IL-1α	Interleukin-1 Alpha
IL-1β	Interleukin-1 Beta
IL-6	Interleukin 6
LDL	Low Density Lipoprotein
MMP	Matrix Metalloproteinase
PC	Positive Cell Ratio
RAGE	Receptor for Advanced Glycation End Products
ROS	Reactive Oxygen Species
SOD	Superoxide Dismutase
TNF-α	Tumor Necrosis Factor-Alpha
ZO-1	Zonula Occludens 1
